# Medical Cost of Colorectal Cancer Services in Serbia Between 2014 and 2017: National Data Report

**DOI:** 10.3389/fphar.2019.00526

**Published:** 2019-05-15

**Authors:** Berislav Vekic, Viktorija Dragojevic-Simic, Mihajlo Jakovljevic, Filip Pilipovic, Radoje Simic, Rastko Zivic, Dragce Radovanovic, Nemanja Rancic

**Affiliations:** ^1^Department of Surgery, Clinical Centre “Dr. Dragisa Misovic”, Belgrade, Serbia; ^2^Faculty of Medical Sciences, University of Kragujevac, Kragujevac, Serbia; ^3^Centre for Clinical Pharmacology, Military Medical Academy, Medical Faculty of Military Medical Academy, University of Defence, Belgrade, Serbia; ^4^Department of Global Health Economics and Policy, Faculty of Medical Sciences, University of Kragujevac, Kragujevac, Serbia; ^5^Institute for Orthopedic and Surgical Diseases “Banjica”, Belgrade, Serbia; ^6^Department for Plastic Surgery, Faculty of Medicine, Institute for Mother and Child Health Care of Serbia “Dr. Vukan Cupic”, University of Belgrade, Belgrade, Serbia; ^7^Clinical Centre “Dr. Dragisa Misovic”, Belgrade, Serbia; ^8^Faculty of Medicine, University of Belgrade, Belgrade, Serbia

**Keywords:** colorectal cancer, medical cost, chemotherapy, diagnostics, pharmacoeconomics

## Colorectal Cancer in Serbia

Colorectal cancer has been a health burden for decades. Although it is considered to be a disease of the developed world, the incidence rate of CRC has been on the rise in developing countries as well (Favoriti et al., 2016; Douaiher et al., 2017). According to the World Health Organization GLOBOCAN database (Bray et al., 2018), is the third most commonly diagnosed cancer and the fourth main cause of cancer death in the world, accounting for 881,000 deaths in 2018. Colorectal cancer also causes substantial morbidity and mortality in Serbia. According to the same database, the number of new cases of in 2018 was 6,049 (12.6% of any form of cancer), while the number of the deaths caused by was 3,187 (2.9% of all cancer-related deaths) (The Global Cancer Observatory, 2018).

Adequate surveillance of CRC occurrence and outcomes is essential for developing effective control programs. The prognosis is strongly related to the stage at the time of diagnosis because late-stage CRCs are associated with more intensive treatments, increased morbidity, and lower survival (La Vecchia et al., 2010; Brenner et al., 2012; Maringe et al., 2013; Favoriti et al., 2016). The reported variability in stage distribution may be due to differences in the timing of diagnosis (early vs. late) and in the thoroughness of staging procedures. This is important because patients diagnosed at an earlier stage are more likely to undergo successful resection and may end up cured. However, direct medical cost very much depends on the initial (T—primary tumor, N—regional lymph nodes, M—distant metastasis) classification stage (Kriza et al., 2013). Among the four stages, the stage I disease is the least costly, whereas stage III is the most costly due to the high cost of biological agents (Kriza et al., 2013). In our country, most of the expenses in terminal stages result from inpatient care and administration of chemotherapy (Kovačević et al., 2015a). Although the use of monoclonal antibodies (mAbs) can significantly extend survival, it is associated with substantial additional cost (Jakovljevic et al., 2014). In addition to expensive treatment options, diagnostic imaging and invasive radiology procedures are further increasing the cost in our region (Jakovljević, 2013; Ranković et al., 2013).

The Serbian health system is facing an additional challenge due to a steep rise in the number of people diagnosed with CRC. From 1997 to 2007, the incidence of CRC increased by 24.6% (Knezevic, 2009). Since this trend will, without doubt, stress the National Health Insurance Fund (NHIF) of Serbia, the objectives of this study were to identify different types of medical services provided to CRC patients and the associated expenditures in order to estimate the total medical cost at the national level between 2104 and 2017. This information is expected to assist healthcare decision planners and policymakers on how to allocate resources optimally.

## Data Report Method

This is a retrospective, observational, descriptive study of different medical expenditures accrued by the patients with CRC in Serbia during the 4-year period. Included in the study were records of all patients with CRC who received medical care at any Serbian hospital regardless of the stage of CRC at the time of diagnosis or treatment.

The main source of information for this study was the registry maintained by the NHIF, which is the government-run health insurance program (https://www.eng.rfzo.rs/index.php). The authors were provided with the data limited to patients with CRC (personal health identifiers removed), which contained information on demographics, medical services provided and the associated expenses (archived at https://figshare.com/account/articles/7660853). The medical services were split into the procedures and expenditures associated with diagnosis, therapy, inpatient/outpatient care, physician examination, preparation and administration of drugs, nursing care, and other related medical services. Note that in Serbia, chemotherapy and radiotherapy can be administered only in the public hospitals and are fully covered by the NHIF, ensuring the validity of our data. The out-of-pocket expenses (OTC preparation, dietary supplements, vitamins, minerals, etc.), loss of productivity-related cost and cost associated with premature death were not available and are beyond the scope of this report.

The use and cost of chemotherapy (including conventional cytotoxic drugs and mAbs) were derived from the publication “Marketing and Consumption of Medical Products for Human Use,” published annually by the Drugs and Medicines and Medical Devices Agency of Serbia (2015–2017 editions). The annual financial reports for the years 2014-2016 were also available therein (accessed at https://www.alims.gov.rs/latin/o-agenciji/publikacije/). The data on the realized market consumption of medication were divided according to the Anatomical Therapeutic Chemical (ATC) Classification System and the international non-proprietary name (INN). The information included in the analysis was pharmaceutical formulations, medication doses and packaging, and defined daily dose (DDD) of drug per 1,000 inhabitants per day. Finally, the consumed drug quantities (packages or DDD) were multiplied by the respective unit prices and summed into total national drug consumption.

The principles of ICH Good Clinical Practice were strictly followed and the approval from the Ethics Committee was obtained (Approval No 26/04/17 for the study protocol No MFVMA/12/17-19, entitled: Cost-effectiveness and cost-utility analysis of CRC treatment and budget impact analysis from the perspective of the patient, hospital and third-party payer).

## The Cost of Colorectal Cancer in Serbia

The number of patients with CRC in Serbia between 2014 and 2017 ranged from about 20 thousand to 21 thousand (Table 1). More specifically, we found a steady increase from 2014 to 2017, with 803 cases more in 2017 than in 2014 (4% increase). Based on the NHIF data for the period 2014–2018, the 5-year prevalence rate of CRC in Serbia was estimated at 17.82/10,000 inhabitants (15,611 cases among 8,762,022 inhabitants). Based on the recently published estimates of CRC incidence and mortality patterns across Europe (Ferlay et al., 2018), the annual number of new cases in Serbia is projected at about 3,700 for men and 2,300 for women. These figures are largely in agreement with the estimates of the World Health Organization GLOBOCAN database (Bray et al., 2018).

**Table 1:** The number of cases of colorectal cancer (CRC) in Serbia, the associated annual cost (€) by healthcare services, and the national economic indicators during the 4-year study period.

	**2014**	**2015**	**2016**	**2017**
**Number of CRC cases**	20,294	20,840	20,841	21,097
**Total healthcare cost**	16,092,057	17,063,551	18,913,701	20,456,361
**Diagnosis cost**	2,914,787	3,242,968	3,424,559	3,697,943
% Total	18.1%	19.0%	18.1%	18.1%
Laboratory	1,019,891	1,174,005	1,266,451	1,393,512
Radiology (classic, MRI, CT, USI, interventional)	709,394	778,402	843,830	914,252
Nuclear medicine	64,543	48,673	55,330	64,872
Endoscopy (diagnosis and therapy)	300,879	265,835	230,391	229,288
Biopsy and surgery (incision)	229,755	227,005	218,046	220,556
Other	590,325	749,048	810,511	875,463
**Therapy cost**	8,889,882	8,940,658	10,348,011	11,376,200
% Total	55.2%	52.4%	54.7%	55.6%
Medication	4,498,386	4,392,597	5,483,289	6,329,859
Medical devices	1,854,445	1,788,037	1,927,796	2,064,889
Surgery	2,022,936	2,166,698	2,205,756	2,244,785
Radiotherapy	276,742	323,498	430,004	404,348
Interventional radiology	1,927	302	1,081	664
Other	235,446	269,526	300,085	331,655
**Inpatient days**	1,920,754	1,927,291	1,924,806	1,938,291
% Total	11.9%	11.3%	10.2%	9.5%
**Outpatient days**	182,625	180,836	198,532	201,964
% Total	1.1%	1.0%	1.0%	1.0%
**Physician examination**	544,597	640,001	642,310	633,877
% Total	3.4%	3.8%	3.4%	3.1%
**Medication preparation/administration**	1,259,979	1,812,227	1,994,169	2,177,385
% Total	7.8%	10.6%	10.5%	10.6%
**Nursing care**	142,424	210,445	236,468	269,477
% Total	0.9%	1.2%	1.2%	1.3%
**Other**	237,009	109,125	144,846	161,224
% Total	1.6%	0.7%	0.9%	0.8%
**GDP (Billions US$)**	44.421	37.160	38.300	41.432
**PHE (%GDP)**	9.842	9.405	10^*^	10^*^
**CRC cost (%PHE)**	0.49	0.65	0.55	0.56

*The relative cost is expressed as a percent of the total healthcare cost (% Total)*.

*MRI, magnetic resonance imaging; CT, computerized tomography; USI, ultrasound imaging; US$, United States dollars; CRC, colorectal cancer; GDP, gross domestic product; PHE, public health expenditure*.

* *Estimated based on the previous year data*.

The total cost of healthcare provided to CRC patients in Serbia was found to be between €16 million and €20.5 million (Table 1) and is clearly rising (27% from 2014 to 2017). In the US, under the best-case scenario, the cost of CRC in 2010 was estimated at $14.14 billion (the second highest among all cancers) and projected to rise to $17.41 billion in 2020, a 23% increase (Mariotto et al., 2011). In addition, CRC bore the highest cost in the initial phase of care because increased use of targeted chemotherapies increases the cost of treatment more rapidly in comparison to the cost of other medical services (Mariotto et al., 2011). In the EU, the overall CRC cost in 2009 was €13.1 billion, accounting for 10% of cost of all cancers (third most expensive following the lung cancer and breast cancer) (Luengo-Fernandez et al., 2013). Considering healthcare cost only, CRC came at the second place, amounting to €5.57 billion (11% of all cancer-related health-care expenditures), just behind the breast cancer.

Our results also show a strong positive relationship between the CRC incidence, CRC health-care expenditures and national income in Serbia. More specifically, our national gross domestic product (GDP, in $) was on the decrease between 2014 ($44.4 billion) and 2017 (41.3 billion), while the public health expenditures (PHE, expressed as a percentage of GDP) remained relatively constant (9.4% to estimated 10%). Considering that the total CRC medical cost increased between 2014 and 2017, CRC expenditures accounted for a greater proportion of PHE over time, from 0.49% in 2014 to 0.56% in 2017 (Table 1, bottom).

We further analyzed the expenditures associated with the diagnosis, therapy, inpatient/outpatient care, physician examination, drug preparation/administration, nursing care, and other services. The NHIF database breaks down diagnostic services into the laboratory, radiology services (classic, magnetic resonance imaging, computed tomography scan, ultrasound imaging, interventional), nuclear medicine, endoscopic services (examinations and treatments), biopsy (including surgery), and other services. We found different trends regarding the cost of these services—the increasing cost of laboratory, radiology, and other services, the relatively steady cost of nuclear medicine and biopsy/surgery services, and the decreasing cost of endoscopy services (Table 1). The cost of CRC diagnosis accounted for about 18% of the total CRC cost and this remained steady between 2014 and 2017 (Table 1). The laboratory portion of the diagnostic cost was the highest (6.6% of the total CRC cost annually, on average). This is somewhat lower than 7.7% laboratory cost reported in our previous study that investigated the cost of end of life medical care for advanced stage cancer patients treated in the tertiary care university hospital in Serbia (Kovačević et al., 2015a). Diagnostic imaging and nuclear medicine diagnosis and therapy amounted up to 4.8% of the total CRC cost annually, an average. The study performed in 2010–2011 at the university healthcare hospital in Belgrade, Serbia, showed that the cost of diagnostic imaging per patient was on average 8.7% of the total CRC cost (Kovačević et al., 2015a). In our previous study that encompassed the network of hospitals in central Serbia, the mean cost per patient was only somewhat smaller-6.5% of the total CRC cost (Dagovic et al., 2014). Finally, taking into consideration this report as well as the previous results on the utilization pattern of radiology services and associated expenditures (Jakovljević et al., 2013, Dagovic et al., 2014; Kovačević et al., 2015a), it appears that nowadays clinicians in our country better adhere to clinical practice guidelines and more rationally order diagnostic procedures for CRC.

We found that the relative cost of inpatient days for CRC was on the continuous decline, from 11.9% in 2014 to 9.5% in 2017, while the cost of outpatient days was rather steady (1.1% in 2014, 1.0% in 2017) (Table 1). The physician examination cost began to drop in 2015, from 3.8 to 3.1% in 2017. Conversely, the relative cost of nursing care was on the rise, from 0.9% (2014) to 1.3% (2017). The cost of drug preparation and administration followed a similar increasing trend (7.8% in 2014, 10.6% in 2017). Collectively, the cost of physician services, inpatient days, nursing care and drug preparation and administration amounted up to 26.9% of the total CRC cost.

The above figures significantly contribute to the findings that oncology services in our country account for the largest proportion of the overall direct medical cost, somewhere between 15.3% (Dagovic et al., 2014) and 21.1% (Kovačević et al., 2015a). This trend is concerning since the overall financial burden of cancer care has also been on the rise over the past decade (almost one-third increase in domestic currency from 2007 to 2010) (Radovanovic et al., 2011). The cost of cancer care in the US in 2010 was estimated to range from $124.6 billion to $137.4 billion (Bradley et al., 2008; Warren et al., 2008; Mariotto et al., 2011), with the projected increase until 2020 of 27% in the best case scenario and 39% in the worst case scenario. In the EU, the total economic cost of cancer care was more than €126 billion in 2009, while the health-care cost was €51.0 billion and accounted for 40% of the total EU health-care expenditures (Luengo-Fernandez et al., 2013). Moreover, inpatient care accounted for more than 50% of all cancer-related healthcare cost and 73% of the CRC-related healthcare cost (Luengo-Fernandez et al., 2013).

## The Cost Structure of Colorectal Cancer Therapy

We found that the overall cost of CRC therapy (medication, medical devices, surgery, radiotherapy, interventional radiology, other services) was quite high, ranging from 52.4% (2015) to 55.6% (2017) of the total CRC cost in Serbia (Table 1).

The relative cost of medication was particularly high, accounting from 25.7% (2015) to 30.9% (2017) of total CRC cost. In our previous studies, anti-cancer medication accounted for 42.5% (Kovačević et al., 2015a) to 58% (Dagovic et al., 2014) of the hospital budget. In the EU, medication accounted for 27% of all cancer-related health-care expenditures in 2009 but the figures differed across the countries, from 15% in Lithuania to 61% in Cyprus (Luengo-Fernandez et al., 2013). Such a wide range may be due to the differences in prices of the same drug, different patterns of drug prescription or relatively lower/higher cost of medication vs. other services. In addition, the above discrepancies may well-reflect variations in clinical practice and price setting and reimbursement mechanisms. More comparative investigations are warranted to clarify this issue.

According to the data from the Drugs and Medical Devices Agency of Serbia, the relative cost of conventional cytotoxic drugs was steadily decreasing and amounted to 22.8, 12.4, 12.0, and 3.0% of all cancer-related drugs in 2014, 2015, 2016, and 2017, respectively. The same data also indicate that the relatively lower cost of conventional cytotoxic drugs is likely due to the relatively higher cost of mAbs (62% to 67.2 from 2014 to 2016). This trend remained largely the same if expressed in euros, except in 2017, when the expenditures for all cytotoxic drugs significantly increased. Based on the same source, the apparent decrease in the expenditures for cytotoxic drugs from 2014 to 2016 was mainly due to the reduced quantities sold rather than decreased prices. For example, as to the cytotoxic drug fluorouracil, the total price decreased for 20.1% from 2014 to 2016 while the number of packages sold went down twice as much (40.7%).

One of the explanations for the above results may be a disturbed process of public procurement, as previously noted at the time of drug tendering process in a tertiary healthcare hospital (Milovanovic et al., 2004). In Serbia, all healthcare purchases are governed by the Law on Public Procurement (https://www.paragraf.rs/propisi/zakon_o_javnim_nabavkama.html), and there are no separate laws that govern the purchases of medication and medical devices. Within this law, the NHIF outlines the so-called “framework agreements,” whereas the healthcare institutions enter into individual agreements. Therefore, the public procurement procedures, at the national level, are resource-consuming, laborious and could be improved, as commonly seen in the countries undergoing a socio-economic transition, like Serbia.

The cost of mAbs dominated other cancer-related expenses. They accounted for 11.3, 12.2, 15.0, and 15.2% of all cancer-related expenses in 2014, 2015, 2016, and 2017, respectively (Tables 1 and 2). Despite an apparent clinical benefit of mAbs for CRC patients, such as longer life expectancy when bevacizumab and cetuximab are added to the common chemotherapy protocols, this comes at a substantial additional cost (Kabbinavar et al., 2005; Van Cutsem et al., 2011; Jakovljevic et al., 2014; Kovacevic et al., 2015b). Not only that mAbs cost more than conventional cytotoxic drugs, the other cost (i.e., nursing care, laboratory tests, physician consults) associated with their administration is also higher (Jakovljevic et al., 2014; Kovacevic et al., 2015b), likely due to more complex drug preparation protocols, more frequent adverse events, and the need for additional follow-up visits. This is somehow in line with the recent report that the annual cost of mAb therapies in the US is about $100,000 higher in oncology and hematology than in other patient groups (Hernandez et al., 2018). Our findings are of great relevance for Serbia, where the value-based turnover of mAbs prescribed in oncology grew almost twenty times over only 9 years, from just over €1 million in 2004 to nearly €20 million in 2012. Therefore, the policymakers should take this into account in order not to cross the upper limits of affordability in our middle-income South-Eastern European economy (Jakovljevic, 2014; Kovacevic et al., 2014, 2015b).

**Table 2:** Total expenditures (€) for chemotherapy in the colorectal cancer patients in Serbia during the 4-year study period by different types of medication used.

	**2014**	**2015**	**2016**	**2017**
**Total chemotherapy**	2,844,126	2,624,157	3,501,863	4,204,787
**Monoclonal antibodies**	1,817,963	2,081,224	2,845,432	3,110,744
% Total	63.9%	79.3%	81.3%	74.0%
Cetuximab	1,370,023	1,371,128	1,836,775	1,614,367
Bevacizumab	447,940	710,096	1,008,657	1,323,629
Panitumumab	–	–	–	172,748
**Conventional cytotoxic drugs**	1,026,164	542,932	656,430	1,094,043
% Total	36.1%	20.7%	18.7%	26.0%
Fluorouracil	174,611	158,659	155,802	148,743
Capecitabine	464,233	125,886	173,373	149,336
Mitomycin	15,137	14,456	24,556	21,998
Oxaliplatin	201,148	116,689	129,066	485,297
Irinotecan	161,798	122,333	169,230	281,245
Other (epirubicin, doxorubicin, vinblastine, vincristine, dacarbazine)	9,238	4,908	4,402	7,423

*The relative cost is expressed as a percent of total chemotherapy cost (% Total)*.

The mAb bevacizumab was registered for CRC therapy in Serbia for the first time in 2005 whereas cetuximab entered the market in 2009 and panitumumab in 2014. Their use has been recommended by the National Guideline on the Good Clinical Practice for Diagnosis and Treatment of CRC, developed the Commission for the Development and Implementation of Good Clinical Practice Guidelines and issued by the Ministry of Health of the Republic of Serbia (The Republic Commission for the Development Implementation of the Good Clinical Practice Guidelines, 2013, http://www.azus.gov.rs/wp-content/uploads/2011/04/Vodic-za-dijagnostikovanje-i-lecenje-raka-kolona-i-rektuma1.pdf). In general, mAbs, including those for CRC, are cataloged in the special section of the National Drug List, the so-called “List C.” Their use is fully reimbursable by the NHIF but can be prescribed only by oncology specialists for certain indications, specific stages of the disease and exclusively administered in the tertiary care hospitals. For example, bevacizumab is prescribed in the CRC stage IVb when potentially operable metastases are found, predominantly in the liver, as the first line treatment in combination with chemotherapy. However, if metastases are resectable and treated surgically, bevacizumab can be administered after surgery (10 cycles maximum). As far as cetuximab, it is used in the metastatic disease following oxaliplatin and irinotecan chemotherapy but exclusively in patients with a wild type K-Ras gene, performance status 0 or 1, either as monotherapy or in combination with irinotecan. Panitumumab is used under the same conditions but as monotherapy only. Since the KRAS mutations are present in 34.7% of the Serbian CRC population (Jakovljevic et al., 2012), they are not treated with cetuximab and panitumumab but instead with bevacizumab only. This practice can explain the more frequent use of bevacizumab than cetuximab. Considering the increased use of mAbs (bevacizumab and cetuximab), and most often in combination with irinotecan and oxaliplatin, the use of other cytotoxic drugs is on the decrease.

Our study has some limitations. The cost estimates should be considered approximate due to the lack of appropriate population-based patient-level cancer data. The data were limited to the medical cost absorbed by the government-run mandatory health insurance program, which precluded consideration of the out-of-pocket expenses and indirect lost productivity-related expenses. Secondly, the cancer staging data were not available, which is necessary for estimating the service cost. However, this national data report is novel since the previous studies were limited to a small number of patients treated in large tertiary care hospitals. In addition, this data report provides, for the first time, an in-depth analysis of both the total medical cost and the cost of key components of medical services provided to the CRC patients in Serbia.

## Conclusions

The number of cases of CRC in Serbia was found to be on the 4% rise between the years 2014 and 2017, whereas the total cost of medical services rendered to these patients overwhelmingly outpaced this trend (27% increase). Such an upsurge in the total cost was estimated to result in about one-third increase in the public health expenditures going toward the CRC medical services over the 4-year study period. Furthermore, individual components of medical services showed variable but not particularly excessive trends, the most notable being a 5% point increase in the cost of all medication used in patients with CRC. In terms of chemotherapy alone, the absolute cost increased by 47.8% and within it, the relative cost of mAbs went 10-percentage points up vis-à-vis the relative cost of conventional cytotoxic drugs.

The outcomes of this study should provide guidance and assist healthcare decision planners and policymakers in allocating resources optimally. More studies on service utilization patterns and cost of cancer care are greatly needed for the countries in transition, particularly in the Balkan region, because evidence-based estimates are critical for ensuring an adequate return on investments in oncology care.

## Author Contributions

BV, VD-S, and NR designed the study and wrote the draft of the manuscript. FP, RS, RZ, and DR searched the literature and analyzed data. MJ critically reviewed the manuscript for important intellectual content. All the authors listed have made a substantial contribution to the conception, development of methodological approach and interpretation of results, and all approved it for publication.

### Conflict of Interest Statement

The authors declare that the research was conducted in the absence of any commercial or financial relationships that could be construed as a potential conflict of interest.
